# An Inserted α/β Subdomain Shapes the Catalytic Pocket of *Lactobacillus johnsonii* Cinnamoyl Esterase

**DOI:** 10.1371/journal.pone.0023269

**Published:** 2011-08-18

**Authors:** Kin-Kwan Lai, Peter J. Stogios, Clara Vu, Xiaohui Xu, Hong Cui, Sara Molloy, Alexei Savchenko, Alexander Yakunin, Claudio F. Gonzalez

**Affiliations:** 1 Department of Microbiology and Cell Science, Genetics Institute, University of Florida, Gainesville, Florida, United States of America; 2 Banting and Best Department of Medical Research, Structural Proteomics in Toronto, University of Toronto, Toronto, Ontario, Canada; 3 UF Undergraduate Research Program MCB4905, Department of Microbiology and Cell Science, University of Florida, Gainesville, Florida, United States of America; University of Queensland, Australia

## Abstract

**Background:**

Microbial enzymes produced in the gastrointestinal tract are primarily responsible for the release and biochemical transformation of absorbable bioactive monophenols. In the present work we described the crystal structure of LJ0536, a serine cinnamoyl esterase produced by the probiotic bacterium *Lactobacillus johnsonii* N6.2.

**Methodology/Principal Findings:**

We crystallized LJ0536 in the apo form and in three substrate-bound complexes. The structure showed a canonical α/β fold characteristic of esterases, and the enzyme is dimeric. Two classical serine esterase motifs (GlyXSerXGly) can be recognized from the amino acid sequence, and the structure revealed that the catalytic triad of the enzyme is formed by Ser_106_, His_225_, and Asp_197_, while the other motif is non-functional. In all substrate-bound complexes, the aromatic acyl group of the ester compound was bound in the deepest part of the catalytic pocket. The binding pocket also contained an unoccupied area that could accommodate larger ligands. The structure revealed a prominent inserted α/β subdomain of 54 amino acids, from which multiple contacts to the aromatic acyl groups of the substrates are made. Inserts of this size are seen in other esterases, but the secondary structure topology of this subdomain of LJ0536 is unique to this enzyme and its closest homolog (Est1E) in the Protein Databank.

**Conclusions:**

The binding mechanism characterized (involving the inserted α/β subdomain) clearly differentiates LJ0536 from enzymes with similar activity of a fungal origin. The structural features herein described together with the activity profile of LJ0536 suggest that this enzyme should be clustered in a new group of bacterial cinnamoyl esterases.

## Introduction

Hydroxycinammates are natural phenolic compounds with a widespread distribution throughout the plant kingdom. These phytophenols are naturally present in the form of monophenols or polyphenols and participate in the formation of macromolecular structures in plant cells. Gastrointestinal absorbable monophenols are particularly interesting to health researchers due to their innate ability to work as free radical scavengers, anti-inflammatory supplements, and immunostimulants [1]–[3]. The health benefits associated with the consumption of natural phenolics are extensively documented and supported by results obtained both *in vitro* and *in vivo* [4], [5]. The most abundant bioactive monophenols present in a balanced human diet are ferulic, caffeic, *p*-coumaric, and sinapic acids [6]. Although the structures of monophenols are similar, the major biochemical differences among them are due to the presence or absence of hydroxyl and/or methyl functional groups attached to the aromatic ring. Monophenols are frequently ester-conjugated to aromatic organic acids to form polyphenols such as oleuropein, chlorogenic acid, or rosmarinic acids, which are present in the diet [7], [8].

Dietary phytophenols can exert action locally immediately following their absorption and act systemically following distribution by the circulatory system [9]. In fact, some of these bioactive compounds can pass the blood brain barrier to reach the central nervous system. Nevertheless, the primary absorption of these compounds at the gastrointestinal level is extremely limited. It was experimentally demonstrated that the monocarboxylic acid transporter is the molecular system capable of mediating cellular uptake of monophenols, such as caffeic and ferulic acids [10]. This system does not have an affinity for esterified polyphenols, since the carboxylic ester group of polyphenols interferes with the recognition system of this specific active transporter. Consequently, these low molecular weight polyphenols, such as rosmarinic acid, are rarely absorbed by paracellular diffusion [9]. In this context, the intestinal hydrolysis of the ester bond is necessary for improving the absorption of bioactive monophenols to maximize their beneficial systemic effects on the host.

However, humans do not synthesize enzymes with the cinnamoyl/feruloyl esterase activity required to break down these ester bonds and release the absorbable bioactive moieties efficiently [11]. It is well known that the cinnamoyl or feruloyl esterase activity present in the human intestinal lumen is produced only by the colonic microbiota [12], [13]. The first cinnamoyl esterase purified from a bacterium commonly found in human colonic microbiota (*Lactobacillus johnsonii*) was recently purified and biochemically characterized in our laboratory [14]. Interestingly, the producer strain, *L. johnsonii* N6.2, was naturally abundant in animal models that are genetically predisposed to develop autoimmune diabetes but do not display the characteristic symptoms of the disease [15]. A subsequent study involving feeding the autoimmune diabetes animal models with *L. johnsonii* N6.2 demonstrated a decrease in the intestinal oxidative stress and a better survival rate [16]. Even though direct involvement of enzymatic activity was not investigated in that publication, the results could be linked to the antioxidative effects of phenolic compounds released from food components by bacterial cinnamoyl esterases.

Cinnamoyl esterases are classical members of the α/β fold structural family. Due to their application in industrial hemicellulose saccharification processes, several fungal enzymes have been biochemically and structurally studied [17]–[19]. In contrast, few bacterial cinnamoyl esterase structures are publicly available. We previously showed that LJ0536, a cinnamoyl esterase purified from *L. johnsonii* N6.2, is active towards a variety of substrates including short acyl chain aliphatic esters and phenolic esters [14]. Herein, we present the crystal structure and mutational analysis of LJ0536. A catalytically inactive Ser_106_Ala mutant was co-crystallized with three different substrates: ferulic acid, ethyl ferulate, and caffeic acid. Co-crystallization results are discussed in the light of enzymological data collected from assays using site-directed mutants. An inserted α/β subdomain was identified as a prominent structure necessary for phenolic ring binding and the formation of the catalytic pocket. Multiple structurally homologous enzymes were identified in the public databases, and many of these contain inserted subdomains of similar size. However, only the Est1E feruloyl esterase of *Butyrivibrio proteoclasticus* [20] contains an inserted α/β subdomain with the same secondary structure and architecture as the insert in LJ0536. We conclude that, despite the presence of the inserted subdomain and nearly identical overall scaffold, the enzymes discriminate substrates through specific features of their active sites.

## Results

### General architecture of the LJ0536 structure

**Figure 1:**
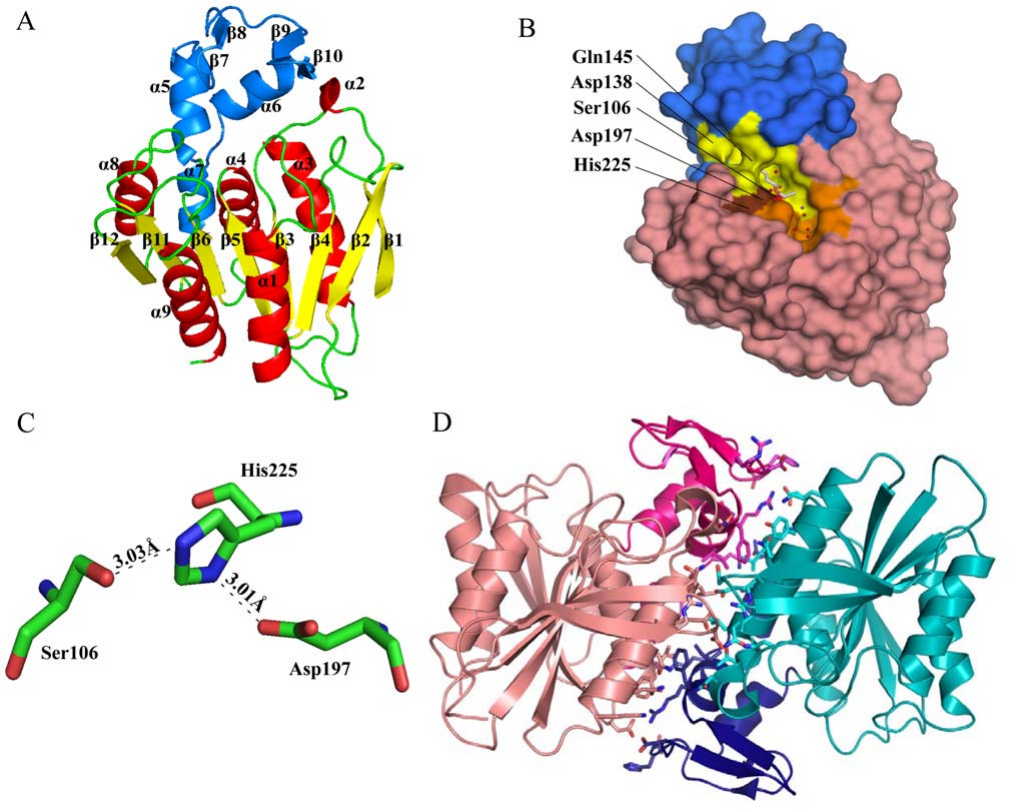
Cartoon and surface representations of LJ0536. (A) Cartoon representation of a single chain of LJ0536. The enzyme displays a classical α/β hydrolase fold as the main protein scaffold and consists of 8 central strands with a left-handed superhelical twist. The central β-sheet core is colored yellow. α–helices are colored red. The inserted α/β subdomain is colored blue. (B) Surface representation of a single chain of LJ0536 Ser_106_Ala mutant (from ethyl ferulate complex Form II). The structure of ethyl ferulate is shown as a stick figure. The inserted α/β subdomain is colored blue. Ser_106_ is colored red. Asp_197_ and His_225_ are colored brown. Other residues that line the ligand binding pocket (have any atom within 4.5 Å of the substrates) are colored yellow. The unoccupied pocket that likely accommodates the quinic acid moiety of chlorogenic acid is colored orange. Water molecules are colored in red and sodium in green. (C) The coordination of catalytic residues of LJ0536. The catalytic triad is composed of Ser_106_, His_225_, and Asp_197_. (D) Cartoon representation of dimeric LJ0536. The two chains are colored in pink or cyan, with the inserted α/β subdomains colored magenta and dark blue, respectively. Amino acids involved in the dimer interface (residues with any atom within 3.8 Å of any atom in the partner chain) are shown in stick representation.

The apo-LJ0536 structure was crystallized in the presence of protease, which facilitated successful crystal growth (the mutant Ser_106_Ala was also crystallized in the presence of protease). The resulting fragment included a loss of four C-terminal residues, such that nearly the entire full-length protein was visible in the electron density map (1–245, plus the five residues from the expression tag). The structure was solved to 2.35 Å (Fig. 1) using Molecular Replacement (MR) with Est1E (PDB:2WTM). Crystallization and diffraction statistics are summarized in Table 1. The native molecular weight was determined to be 46.0 kDa by gel filtration (monomeric apparent molecular weight = 27.6 kDa). The molecule displayed a classical α/β hydrolase fold [21]. The overall structure of LJ0536 consisted of a central β-sheet composed of seven parallel (β_1_, β_3_, β_4_, β_5_, β_6_, β_11_, β_12_) and one antiparallel β-strand (β_2_). This central β-sheet shows a left-handed superhelical twist with an approximate 120° angle from β_1_ to β_12_. It is flanked by five α–helices with two α–helices (α1, α9) externally located and three α–helices (α3, α4, α8) internally located towards the dimer interface. The asymmetric unit of the apo-LJ0536 crystal contained two protein molecules that formed an extensive interface (Fig. 1D). This interface is formed by α_4_, α_6_, and β_1_. As calculated by the PDBe PISA (Protein Interactions, Surfaces, Assemblies) server, which identifies interfaces between proteins by measuring their buried surface area, the dimer interface comprises 34 and 37 residues of chain A and chain B, respectively, burying a total of 2373 Å^2^ between the two chains. The interface is primarily hydrophobic (18/37 residues from chain B), although there are six salt-bridges as well. Due to these characteristics, this interface is presumed to be the dimer interface of the protein. A sequence of 54 amino acids (Pro_131_ to Gln_184_) forms an inserted α/β subdomain that is between the β_6_ and β_11_ strands. This insertion is composed of two short β-hairpins (β_7_/β_8,_ β_9_/β_10_) and three α–helices (α_5_, α_6_, α_7_). The two β-hairpins are projected towards the entrance of the catalytic site.

**Table 1:** X-ray diffraction and structure determination statistics.

PDB code	3PF8	3PF9	3S2Z	3PFB	3QM1	3PFC
Enzyme	LJ0536 (wild-type)	LJ0536 S106A	LJ0536 S106A	LJ0536 S106A	LJ0536 S106A	LJ0536 S106A
Ligands	None	None	Caffeic acid (from soak of chlorogenic acid)	Ethyl ferulate, Form I	Ethyl ferulate, Form II	Ferulic acid
**Data collection**						
Wavelength (Å)	*Cu-Kα* 1.54178	*Cu-Kα* 1.54178	*Cu-Kα* 1.54178	*Cu-Kα* 1.54178	*Cu-Kα* 1.54178	*Cu-Kα* 1.54178
Resolution (Å)	50.0 – 2.35	50.0 – 1.75	50.0 – 1.75	50.0 – 10.0.58	23.80 – 1.82	50.0 – 1.75
Space group	R3_2_	C222_1_	C2	C2	C222_1_	C222_1_
Cell dimensions						
a, b, c (Å)	149.9, 149.9, 130.3	72.7, 85.7, 81.9	72.3, 84.2, 87.6	72.3, 83.9, 88.9	71.9, 85.4, 81.1	72.0, 85.4, 81.0
a, b, g (°)	90, 90, 120	90, 90, 90	90, 97.6, 90	90, 98.2, 90	90, 90, 90	90, 90, 90
Number of observed reflections	143192	140545	208914	101781	139955	146324
Number of unique reflections	23389	26002	51281	46265	22853	25396
R_sym_	0.105 (0.442)^a^	0.057 (0.460)^b^	0.047 (0.462)^c^	0.046 (0.260)^d^	0.048 (0.327)^e^	0.058 (0.484)^f^
I/σI	13.44 (4.81)	37.87 (3.75)	21.86 (2.57)	31.21 (3.09)	27.56 (3.18)	22.20 (2.88)
Completeness (%)	99.0 (100.0)	99.1 (96.1)	99.7 (97.6)	73.1 (21.6)	100 (99.9)	99.5 (94.0)
Redundancy	6.1 (5.5)	5.4 (4.1)	4.1 (3.3)	2.0 (1.3)	6.1 (4.8)	5.8 (5.1)
**Refinement**						
Programs	Refmac, PHENIX, BUSTER	Refmac	Refmac	PHENIX, Refmac	PHENIX	PHENIX
Resolution (Å)	31.49 – 2.34	50.0 – 1.75	50.0 – 1.76	44.20 – 1.58	23.07 – 1.82	17.65 – 1.75
Number of reflections: working, test	22192, 1194	23365, 1310	48659, 2615	47130, 2673	21234, 1119	23466, 1237
R_work_/R_free, 5%_	22.3/29.9 (27.3/36.5)	14.1/19.9 (23.6/30.7)	14.3/20.8 (19.0/28.2)	21.1/30.4 (30.2/36.2)	14.7/19.1 (22.8/26.7)	14.7/17.9 (23.6/26.1)
No. atoms						
Protein	3836	1988	3935	3939	1991	1964
Ligands	N/A	N/A	26	32	16	14
Solvent	3	4	16	20	61	17
Water	146	275	432	907	173	224
Average *B*-factors						
Protein	56.7	29.9	33.9	23.6	19.4	22.9
Ligand	N/A	N/A	35.9	33.0	19.1	21.8
Solvent	39.2	41.9	58.0	26.0	55.9	49.4
Water	44.9	43.3	46.7	39.2	31.5	24.4
R.m.s. deviations						
Bond lengths (Å)	0.010	0.025	0.023	0.021	0.016	0.016
Bond angles (°)	1.23	1.843	1.812	1.933	1.710	1.611
Ramachandran analysis						
Most favoured (%)	86.4	89.1	89.3	88.4	90.8	90.7
Additionally favoured (%)	12.0	9.5	9.1	10.0	7.3	7.9
Generously favoured (%)	0.9	0.5	1.1	1.1	1.4	1.4
Disallowed (%)	0.7	0.9	0.5	0.5	0.5	0

a Values in brackets refer to the highest resolution shell of 2.39 – 2.35 Å.

b Values in brackets refer to the highest resolution shell of 1.78 – 1.75 Å.

c Values in brackets refer to the highest resolution shell of 1.78 – 1.75 Å.

d Values in brackets refer to the highest resolution shell of 1.61 – 1.58 Å.

e Values in brackets refer to the highest resolution shell of 1.85 – 1.82 Å.

f Values in brackets refer to the highest resolution shell of 1.78 – 1.75 Å.

### The enzyme hydrolytic center

The presumed catalytic pocket resembles an open canal-like feature, which has the shape of a boomerang that ends in a hydrophobic pocket buried between the α_5_ and α_6_ of the inserted α/β subdomain (Fig. 1A and 1B). Using the Ser_106_ as the center of the boomerang, one of the two clefts of the catalytic pocket is approximately 13 Å long and 7 Å, and it is large enough to accommodate the aromatic acyl group of the substrate. The two protruding hairpins from the inserted α/β subdomain decorate the entrance of this cleft and form the “roof” of the catalytic compartment. The other cleft is about 12 Å long and can accommodate the alkoxyl group plus additional atoms from larger substrates.

The catalytic pocket contains the presumed catalytic triad composed of Ser_106_, His_225_, and Asp_197_ (Fig. 1B and 1C) with the catalytic serine residue located at the nucleophilic elbow formed between β_5_ and α_4_. The oxyanion hole formed by the backbone nitrogen atoms of Phe_34_ and Gln_107_ is buried towards the base of the inserted α/β subdomain. Ser_106_ takes part in a classical Gly_104_-X-Ser_106_-X-Gly_108_ serine esterase motif previously identified by analysis of the linear sequence [14]. In addition, a potential second motif Gly_66_-X-Ser_68_-X-Gly_70_ was identified in a highly conserved region (Fig.S1) that is 18 Å from the first catalytic motif (Fig.S2). We validated that the active catalytic triad is Ser_106_, His_225_, Asp_197_, since the Ser_106_Ala and Asp_197_Ala mutants displayed no catalytic activity (Table 2). The His_225_Ala mutant retained approximately one-fifth of the wild-type activity (as measured by k_cat_), but nonetheless the activity is drastically hampered. In contrast, the structure revealed no formation of a catalytic pocket associated with Ser_68_. The potential triad (Ser_68_, His_32_, Asp_61_) is not in the correct orientation and Ser_68_ is not located at the sharp turn of any nucleophilic elbow (Fig.S2). Circular dichroism analysis of the Ser_68_Ala mutant confirmed a significant change in the overall secondary structure of the protein (Fig.S3). This analysis indicates that the activity of this mutant is affected by an overall change in the protein structure rather than by a direct change of a catalytic residue.

**Table 2:** Saturation kinetic parameters of LJ0536 mutants using 4-nitrophenyl butyrate as substrate.

Mutants (description)	V_max_ (µmol/mg/min)	K_m_ (mM)	k_cat_ (s^−1^)	k_cat_/K_m_ (M^−1^s^−1^)
H32A (inactive motif)	0.09±0.02	0.13±0.08	0.04	3.18 E+02
D61A (inactive motif)	0.00±0.00	0.00±0.00	0	0
S68A (inactive motif)	0.47±0.04	0.08±0.02	0.22	2.70 E+02
D138A (substrate-interaction)	1.23±0.08	0.22±0.03	0.57	2.57 E+03
Q145A (substrate-interaction)	2.05±0.10	0.29±0.04	0.94	3.25 E+03
S106A (active motif)	0.00±0.00	0.00±0.00	0	0
D197A (active motif)	0.00±0.00	0.19±0.06	0	0
H225A (active motif)	0.65±0.06	0.19±0.04	0.3	1.57 E+03
Delta_147–173_ (subdomain)	0.28±0.03	0.65±0.12	0.13	1.98 E+02
WT (control)	3.34±0.15	0.16±0.02	1.53	9.59 E+03

### Analysis of the crystal structures of Ser_106_Ala-substrate complexes reveals residues important for substrate binding and catalysis

**Figure 2:**
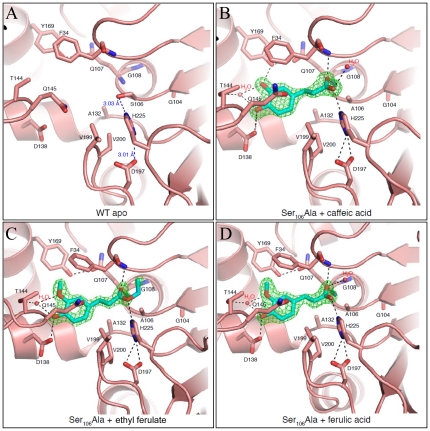
Substrate binding sites of apo WT LJ0536 and Ser_106_Ala bound to various substrates. Substrates are shown in stick representation. Residues that are within 3.5 Å of the substrates are also shown in stick representation. Electron density maps (green) shown are F_o_-F_c_ maps at 3.0 σ calculated after simulated annealing. Relevant hydrogen bond interactions in forming the active site are shown in dashes. Water molecules are colored red. (A) Catalytic site of WT apo LJ0536. Distances between residues of the catalytic triad are labeled (B) Catalytic site of LJ0536 Ser_106_Ala bound with caffeic acid from crystals soaked with chlorogenic acid. (C) Catalytic site of LJ0536 Ser_106_Ala bound with ethyl ferulate. (D) Catalytic site of LJ0536 Ser_106_Ala bound with ferulic acid.

We determined the structure of the active site mutant (Ser_106_Ala mutant) and also co-crystallized this mutant with ferulic acid, ethyl ferulate, or chlorogenic acid (Fig. 2). The Ser_106_Ala-ethyl ferulate complex crystallized in two forms (Form I and Form II with a dimer and a single chain in the asymmetric units, respectively). The two ethyl ferulate crystal forms are nearly identical in structure. The root mean square deviation (RMSD) values of 244 Cα atoms of both chains of Form I onto Form II are 0.25 and 0.3 Å, respectively, and the dimer from Form II is essentially identical to the Form I dimer. We focused our analysis on Form II due to better occupancy of the ligand in the active site. Overall, we observed no appreciable differences in the backbone structure between the apo wild-type (WT) enzyme and the Ser_106_Ala mutant (RMSD of 0.33 Å over all 244 Cα atoms). The ligand binding did not induce major structural changes in the active site, except for a rotamer change in Gln_145_ and a slight rotation of the side chain of His_225_. This created a second hydrogen bond to Asp_197_ (Fig. 2), which is presumably the active conformation of the catalytic triad.

The excellent diffraction of these crystals (resolutions between 1.58 Å–1.75 Å) allowed us to compare the position of different substrates and the conformation of the active site residues (Fig.S4A). Gln_145_ from the inserted α/β subdomain adopted a different conformation, creating a bridge-like structure on top of the catalytic site (Fig. 1B). This feature, along with the side chains of Phe_34_ and Val_199_, limits the size of substrate that can enter the catalytic pocket to 7 Å in width. In all three complexes, the substrates in the catalytic groove were oriented with the aromatic acyl moiety of the carbonyl group bound in the deepest part of the pocket (Fig. 2). The opposite end of the ligands, on the far side of the ester moiety, such as the ethoxyl group of ethyl ferulate, rests on a more solvent-exposed area of the groove and has few interactions with the protein. The electron density for the C2 atom of ethyl ferulate has missing electron density in our structure, which is consistent with this atom being part of the leaving group after hydrolysis. As well, no clear electron density was resolved for the quinic acid moiety of chlorogenic acid (labeled as caffeic acid-bound), perhaps due to residual enzymatic activity, or a lack of productive interactions with the enzyme. The enzyme does have an area of the binding cleft that would accommodate the quinic acid group, or other groups with similar size, formed by the side chains of His_32_, Ala_36_, Thr_40_, Leu_42,_ Leu_43,_ His_105_, and Cys_226_ in the binding cleft (Fig. 1B and Fig.S4B). This pocket is occupied by water molecules and/or sodium ions in each of our structures (Fig. 2).

**Figure 3:**
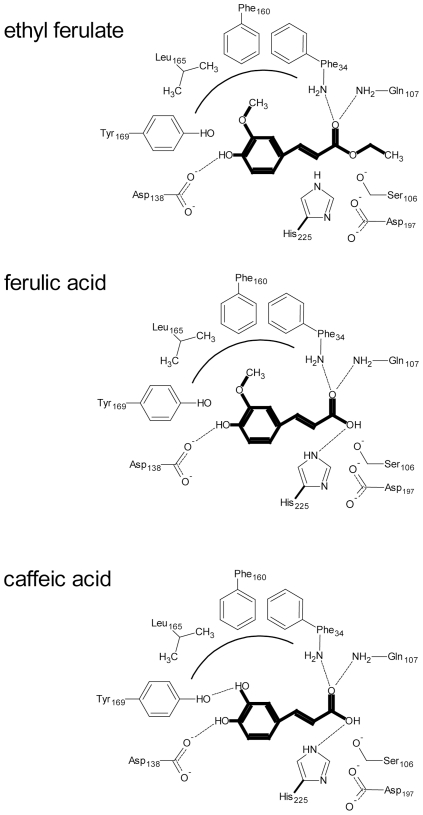
Schematic diagram of LJ0536-substrate interactions. The substrate and products are highlighted in bold. Dotted line represents hydrogen bonds. Curved line represents the hydrophobic cavity created by Phe_34_, Phe_160_, and Leu_165_. The 3-methoxy group of the ferulic ring is oriented towards the hydrophobic cavity. Asp_138_ is hydrogen bonded to the 4-hydroxyl group on the ring of ferulic and caffeic acid. Tyr_169_ is hydrogen bonded to the 3-hydroxyl group of caffeic acid ring. The oxyanion hole is formed by the backbone nitrogen atoms of Phe_34_ and Gln_107_. (A) Ethyl ferulate bound. The distance between the catalytic serine hydroxyl group and the ester carbon is 2.1 Å and 2.3 Å in chain A and chain B, respectively. (B) Ferulic acid bound. The distance between the catalytic serine hydroxyl group and the ester carbon is 2.0 Å. (C) Caffeic acid bound. The distance between the catalytic serine hydroxyl group and the ester carbon is 2.2 Å and 2.0 Å in chain A and chain B, respectively. Note that distances between the catalytic serine hydroxyl group and ester carbons are based on modeling the serine rotamer seen in the WT structure (*t* rotamer, chi angle = −178) onto the structures of the S106A mutant-substrate complexes.

The protein forms extensive hydrogen bonding networks at both ends of the ligands, such that the protein forms a molecular ruler where the distance between the aromatic ring and the site of hydrolysis is constrained by these hydrogen bonds and the position of the catalytic triad. Other than the catalytic residues and the oxyanion hole, the enzyme does not contribute any hydrogen bonds at the end of the ligand with the ester group (Fig. 2 and 3). This suggests that substrate discrimination is accomplished by the hydrogen bonds to the aromatic ring and its substituents. More hydrogen bonds are formed with the phenolic rings of the ligands, including the presence of an ordered water molecule in all of the complexes (Fig. 2). The 4-hydroxyl group (ethyl ferulate, ferulic acid, and caffeic acid) and 3-hydroxyl group (caffeic acid) of the aromatic ring of the substrates are hydrogen bonded to Asp_138_ and Tyr_169_, respectively, from the inserted α/β subdomain at the back of the enzyme cavity. The 3-methoxy (3-hydroxyl in the case of caffeic acid) and 4-hydroxyl groups also interact with an ordered water molecule in all of the complexes. This water is also coordinated by the Oγ1 atom of Thr_144_ from the inserted α/β subdomain. The 3-methoxy group of ethyl ferulate or ferulic acid is accommodated by a small hydrophobic cavity formed by the benzyl moieties of Phe_34_ and Phe_160_, plus the Leu_165_ residue (Fig. 3). The aliphatic chain separating the aromatic ring from the site of hydrolysis is accommodated by hydrophobic side chains (Phe_34_, Ala_132_, Val_199_, and Val_200_). One oxygen atom of the carbonyl group that forms the ester interacts directly with the oxyanion hole formed by the backbone nitrogen atoms of Phe_34_ and Gln_107_, while the other oxygen interacts with His_225_ and an ordered water molecule present in the caffeic and ferulic acid structures (the ethoxy group of ethyl ferulate occupies the space of this water molecule). Due to these interactions, ethyl ferulate rotates slightly and positions the ester bond perpendicular to Ser_106_ at a distance of 2.73 Å. The ester bond is strained from planarity (bond angle of 116°), suggesting that the hydrolytic mechanism involves a tetrahedral enzyme-ester intermediate, typical of esterases. The fact that the ester bond of the ferulic acid product is in a planar configuration that is parallel to the main axis of the groove is further suggestion of this mechanism (Fig. 2C, 2D, and Fig.S4A).

We observed a water molecule 3.3 Å from the non-carbonyl oxygen of the ester bond of ethyl ferulate towards the solvent-exposed face of the pocket. It is possible that this corresponds to the water molecule that is the target for deprotonation by His_225_ to hydrolyze the tetrahedral intermediate between the ligand and Ser_106_, regenerating the enzyme and releasing the product. After hydrolysis, the new hydroxyl group forms a polar interaction with the Nε2 of His_225_.

The caffeic acid moiety of chlorogenic acid adopts a similar position and interaction with the catalytic pocket, as does ferulic acid. However, caffeic acid has two hydroxyl groups in the benzyl ring (positions 3 and 4) which interact with the side chain of Asp_138_ and Tyr_169_ through hydrogen bonding (Fig. 2 and 3). These differences between enzyme and substrate interaction explain the differences in the turnover number previously reported (chlorogenic acid K_cat_ = 28.1 s^−1^; ethyl ferulate K_cat_ = 7.9 s^−1^) [14].

The size of the binding pocket as revealed in the crystal structure helps explain the results of a previous study as to why this enzyme has lower substrate affinity (based on K_m_) with 1/2-naphthyl acetate compared to 1/2-naphthyl propionate and butyrate (1-Naphthyl-acetate: 0.298±0.03 mM. 1-Naphthyl propionate: 0.162±0.01 mM. 1-Naphthyl butyrate: 0.150±0.01 mM. 2-Naphthyl acetate: 0.897±0.22 mM. 2-Naphthyl propionate: 0.225±0.02 mM. 2-Naphthyl butyrate: 0.222±0.01 mM) [14]. It is possible that the size of acetate is not long enough to fully exploit the binding pocket for interactions.

### Site-directed mutagenesis of the inserted α/β subdomain demonstrates a role in substrate preference

**Figure 4:**
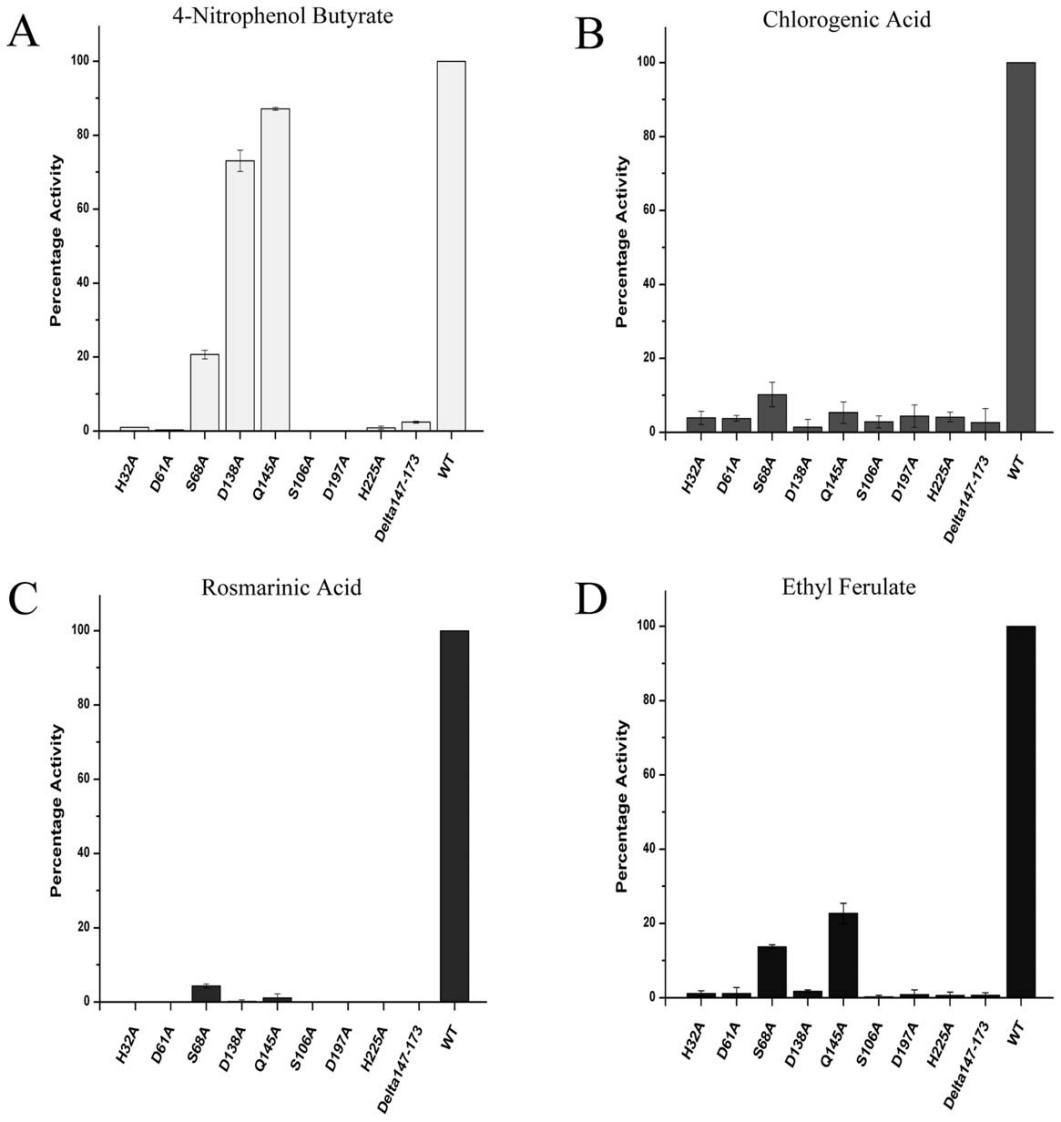
Activity of LJ0536 mutants with (A) 0.1 mM 4-nitrophenyl butyrate. (B) 0.1 mM chlorogenic acid. (C) 0.1 mM rosmarinic acid. (D) 0.1 mM ethyl ferulate. This activity profile shows Asp_138_ and Gln_145_ are the critical residues involved in substrate recognition.

The analysis of the catalytic site indicated that the inserted α/β subdomain from Pro_131_ to Gln_184_ could be important for substrate binding. We hypothesized that this structure is critical for holding the phenolic ring of the phenolic esters in the correct position for catalysis but has a less important role when aliphatic esters are used as the enzyme substrate. We assessed this hypothesis by introducing a dramatic change to the enzyme and expressing a partial deletion mutant of the inserted α/β subdomain. The purified enzyme carrying the deletion Δ_147–173_ showed low activity when 4-nitrophenyl butyrate was used as the model substrate (Table 2). In contrast, no activity was detected with any of the phenolic esters (ethyl ferulate, chlorogenic acid, and rosmarinic acid), even when excessive amounts of enzyme (50 µg/ml) were used in the reaction mixtures and analyzed using HPLC. Although we cannot exclude the possibility that this mutant is misfolded, we can only assume that this deletion does not introduce changes to protein folding based on the residual activity observed from saturation kinetic assay. A deeper analysis of site-directed mutants confirmed the importance of the inserted α/β subdomain in phenolic ester catalysis (Table 2, Fig. 4). Among these mutants, Asp_138_Ala and Gln_145_Ala had the highest impact on the enzymatic activity. A direct comparison using four different substrates at a fixed concentration (0.1 mM) indicated that Asp_138_Ala and Gln_145_Ala showed 73.1±2.8% and 87.6±0.3% activity, respectively, towards 4-nitrophenyl butyrate (Fig. 4). The activity of these mutants dropped to less than 10% when caffeic acid esters (chlorogenic acid and rosmarinic acid) were used as substrates (Fig. 4B and 4C). Interestingly, the mutant Gln_145_Ala retained 21.7±2.8% activity when ethyl ferulate was used as the substrate (Fig. 4D). These results suggested that the residues Asp_138_ and Gln_145_ play a role in interacting with and/or restricting access to the binding pocket for caffeic and feruloyl esters, but not for nitrophenyl-based esters. A possible explanation could be that the orientation of the ester bond in 4-nitrophenyl butyrate is such that in order to maintain the proper orientation in the binding site for catalysis, this substrate would need to be oriented with the 4-nitrophenyl moiety bound in the other pocket of the boomerang-shaped binding canal. This hypothesis would have to be tested by mutation, such as to Thr_40_ or His_105_. These results together with the crystallographic data indicated that Asp_138_ and Gln_145_ from the inserted α/β subdomain are important in recognizing the caffeic and feruloyl esters.

### Sequence and structural comparisons of LJ0536 with the α/β hydrolase superfamily

We undertook PSI-BLAST searches of Genbank [22] to better understand the genomic distribution of the α/β subdomain of LJ0536 and to identify whether it is unique to this enzyme. We utilized the PSI-BLAST algorithm, which develops position-specific scoring matrices to identify remote sequence homology [23]. We reasoned that if the α/β subdomain is unique to LJ0536 and closely-related enzymes, PSI-BLAST searches would not reveal significant matches to other types of hydrolase enzymes in other genomes. We also reasoned that conversely, searches using the full-length sequence of LJ0536 should reveal a wide range of hydrolase enzymes from diverse genomes. The results matched this reasoning very well. A PSI-BLAST search with the full-length sequence retrieved various α/β fold hydrolases upon the first iteration, such as esterases, thioester hydrolases, peptidases, and lipases, from Gram positive or negative bacteria, archea, and plants (data not shown). Conversely, a PSI-BLAST search with residues 131–184 of LJ0536 did not retrieve the same range of hydrolases as the search with the full-length sequence, even after the search reached convergence after six cycles. The ranked order of the results is *Lactobacillus* hydrolases, followed by cinnamoyl hydrolases from *Bacteroides* and *Actinobacteria*. The sixth iteration retrieved a match with Est1E from *Butyrivibrio proteoclasticus*. Therefore, we concluded that the α/β subdomain is unique to cinnamoyl esterases from *Firmicutes*, *Bacteroides*, and *Actinobacteria*.

We were also interested in structural similarity searches to gain insight into the conservation of the LJ0536 structure and the α/β subdomain. A structural similarity search using the Dali Database [24], which calculates intramolecular distances in a structure and then compares the result with similar calculations performed on the contents of the Protein Databank, identified many proteins with structural homology to LJ0536. This was based on similarity of the overall α/β hydrolase fold. The top matches were Est1E from *Butyrivibrio proteoclasticus* (PDB: 2WTM) [20], human mono-glyceride lipase (PDB: 3JW8 and 3HJU) [25], bromoperoxidase A1 from *Streptomyces aureofaciens* (PDB: 1A8Q) [26], and aryl esterase from *Pseudomonas fluorescens* (PDB: 3HI4) [27]. After structure superposition, these enzymes have a range of sequence identities between 17% and 32%, suggesting a low level of sequence similarity, over nearly the full-length of the LJ0536 structure (220–244 matching Cα atoms). Further down the list of matches, LJ0536 has structural similarity with valacyclovir hydrolase (VACVase) (PDB: 2OCG) [28] with 20% sequence identity over 221 matching Cα atoms (nearly the full-length of both structures).

**Figure 5:**
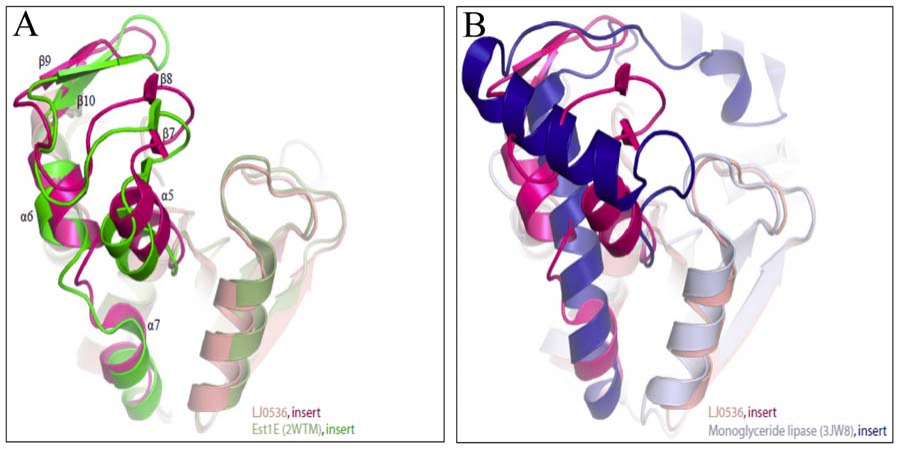
Comparison of the structure of the inserted subdomain. The inserted α/β subdomain of LJ0536 is conserved with (A) Est1E but not conserved with others such as (B) monoglyceride lipase.

Superposition of these structures showed that the enzymes are highly similar in the architecture of the esterase fold, but there is structural variation in the inserted subdomains (Fig. 5). LJ0536 and Est1E superimpose with a RMSD of 0.9 Å, indicating a high level of structural similarity, over nearly the full-length of both structures (231 matching Cα atoms which includes the inserted subdomains). The inserted α/β subdomain of Est1E is highly similar to that of LJ0536, as it contains the same secondary structure topology. However, the other structural homologs listed above have different topologies of their inserted subdomains. These inserted subdomains are always all-α helical, which differs from the mixed α/β LJ0536 insert. For example, while VACVase contains an inserted subdomain, it is comprised of four α-helices. An optimal superimposition of LJ0536 and VACVase resulted when the inserted subdomains were excluded (RMSD of 1.6 Å over 140 matching Cα atoms).

The structural variation in the inserted α/β subdomain is reflected in substrate preferences. Est1E is indeed a closely related enzyme to LJ0536 as seen by its activity towards feruloyl esters [20]. Unlike most of the esterases, VACVase demonstrated high specificity for amino acid esters. Since VACVase is an enzyme of interest involved in prodrug activation, we purified this enzyme and compared its activity in parallel with LJ0536 to assess their substrate preferences. Previously published results [28] were successfully reproduced and VACVase was not active towards 4-nitrophenyl esters, ethyl ferulate, chlorogenic acid, or rosmarinic acid. In agreement with this, despite the fact that LJ0536 has a large range of catalytic specificity, no activities were detected when valacyclovir and L-amino acid benzyl esters were used as substrates for this enzyme.

Considering the sequence, structural, mutational, and substrate preference data, the results suggest the architecture of the inserted α/β subdomain of cinnamoyl esterases is a unique structure that plays a role in conferring substrate specificity towards cinnamoyl/feruloyl esters.

## Discussion

The hydrolytic mechanism of serine esterases is well characterized [29], [30]. However, the mechanism by which this group of promiscuous enzymes recognizes their substrates is still a subject of scientific debate. Thus, the discussion of this work is focused on the analysis of the relationship between the substrates used and the amino acids that form the catalytic pocket of LJ0536.

The overall structural features of *L. johnsonii* cinnamoyl esterase LJ0536 resemble those recently described in Est1E, a predominant esterase encoded in the genome of *Butyrivivrio proteoclasticu*s [20]. However, the specific structural differences in the architecture of the catalytic pocket promote different substrate binding preferences. The differences in the catalytic pocket scaffolds become evident when both protein structures are superimposed. The protruding hairpins of LJ0536 at the entrance of the catalytic groove are slightly shifted (2.30 Å and 1.85 Å) with respect to Est1E. Consequently, the catalytic serine of LJ0536 is more exposed to the solvent. The inserted α/β subdomain of LJ0536 adopts the same conformation in the apo and ligand-bound structures, suggesting that this is a rigid structure. In contrast, it was suggested that the catalytic pocket of Est1E changes conformation upon substrate binding [20]. The pocket flexibility of Est1E is based on the rotation of Trp_160_. When ferulic acid was bound to the catalytic site, the protruding hairpins of Est1E shifted. Trp_160_, which is located on the second protruding hairpin, flips and creates a small hydrophobic pocket, allowing the binding of substrate. The rotation of Trp_160_ on this second hairpin, which corresponds to the hairpin formed between β9 and β10 in LJ0536, forms a tunnel to bury the ferulic acid in the catalytic pocket. In addition, the backbone of Leu_144_ forms a hydrogen bond to the 4-hydroxyl group on the aromatic ring of ferulic acid. This dynamic mechanism is likely not present in LJ0536. Trp_160_ of Est1E corresponds to Phe_160_ of LJ0536, and Phe_160_ adopts the same conformation in the apo and each of the ligand-bound complexes. Leu_144_ of Est1E corresponds to Gln_145_ of LJ0536. The bridge-like structure formed by this residue in LJ0536, which lies on the first hairpin of the inserted α/β subdomain, is present in the same location in both apo and holo structures. Hydrogen bonding in the pocket of LJ0536 is formed between side chains (Asp_138_ and Tyr_169_) and the hydroxyl groups on the aromatic ring of ferulic acid and caffeic acid.

The orientation of substrates in the catalytic pocket of LJ0536 is important for catalysis. The correct conformation is acquired through interaction of the enzyme with the substituents on the aromatic ring on one end of the ligands and with the ester group on the other. These two points of interaction fix the carbonyl group near the oxyanion hole and allow bending of the molecule. Conversely, functionalities beyond the ester group, including the alkoxyl group of ethyl ferulate/ferulic acid and the quinic acid moiety of chlorogenic acid, appear not to play roles in substrate binding. This notion is suggested due to the fact that we did not observe electron density for the quinic acid moiety of chlorogenic acid, although the binding cleft is large enough to accommodate this group. In addition, there was relatively poor density for the alkoxyl group, perhaps due to the lack of direct interaction with the catalytic pocket. The substrate specificity only depends on the type of phenolic acid present in the ester. Binding of the leaving group is not necessary, which results in a poor electron density map of the leaving group. This hypothesis is supported by Faulds *et al.* [18], which showed that crystallization of a S133A AnFaeA mutant (catalytic deficient mutant of feruloyl esterase from *Aspergillus niger*) with feruloylated trisaccharide substrate shows only the ferulic acid moiety but not the carbohydrate moiety. A similar scenario is observed in the crystal structure of a catalytic deficient S172A FAE-XynZ mutant in complex with feruloyl arabinoxylan [31]. Only the ferulic acid is visible in the structure, even though the authors took extra precaution to avoid substrate hydrolysis during crystallization. Both studies lead to the same conclusion that the lack of leaving group in the structure is due to the lack of interaction between the enzyme and the leaving group.

In regards to the catalytic mechanism of LJ0536, the active site is formed by the classical triad of Ser, His, and Asp [32]. The role of His_225_ is to deprotonate Ser_106_ so that Ser_106_ can perform a nucleophilic attack on the carbon atom of the carbonyl group of the substrate, while Asp_197_ stabilizes the protonated His_225_. An intriguing feature of LJ0536 is the presence of a second GlyXSerXGly motif, which is conserved among LJ0536 orthologs. The GlyXSerXGly harboring the catalytic serine was identified at position Gly_104_-Ser_106_-Gly_108_ while the second motif is located at Gly_66_-Ser_68_-Gly_70_. Even though Ser_68_ is exposed to the exterior of the enzyme and the two other conserved His_32_ and Asp_61_ are found in the vicinity of Ser_68_, the orientation of these amino acids makes it impossible to form an active catalytic site. Analysis of the structure suggested that Asp_61_ and Ser_68_ form hydrogen bonds with Val_14_ on β_2_ strand of the central core, and these interactions could play a role in maintaining the proper folding and/or structure of the enzyme. Thus, the drastic decrease in activity detected in the Ser_68_Ala mutant could be related to changes in the overall structure of the protein as suggested by the circular dichroism assay (Fig.S2). We did not observe any evidence, such as internal repeated sequences, that would support gene duplications or recombination during evolution.

Structural similarity searches revealed a number of structural homologs of LJ0536. This included VACVase, which is a biphenyl hydrolase-like protein originally identified from human breast carcinoma and is usually produced in large amounts in the liver. This enzyme was also detected in Caco-2 cells, as well as in the intestinal mucosa [28], [33]. Since this protein could potentially contribute to phytophenol ester hydrolysis in the human intestine and the substrates used herein were not previously assayed, the activities of both enzymes were analyzed in parallel. Our results indicated that, despite the overall structure conservation of the enzymes, the substrate preferences of the enzymes are completely different. This could be due to the structural variations in the inserted subdomain, which our structural superimpositions demonstrated.

A recent review proposes a novel classification scheme for feruloyl esterases [34] based on a combination of several features, such as enzymatic activity, sequence similarity, ligand profile, and structural conservation. Since only a few ferulic acid esterases from bacterial origin have been fully characterized, the classification scheme proposed relies largely on proteins of fungal origin. Neither LJ0536 nor any of its homologs are yet included in any groups in this review [34]. The major differences between the enzymes herein studied and the esterases of fungal origin are related to the architecture of the catalytic pocket and substrate binding. For example, the *Aspergillus niger* AnFaeA (PDB: 2BJH) pocket [19] is a narrow, open cleft formed by a small loop of 23 amino acids and a short α helix. The ferulic acid is wedged between the two walls of the crevice. The hydrophobic stabilization of the aromatic ring in ferulic acid is contributed by the amino acids on one of the walls; the methoxy group decorating the benzyl ring of ferulic acid is oriented towards a small cavity composed of polar amino acids. AnFaeA along with the other fungal structures are clearly different from the inserted α/β subdomain of LJ0536. Consequently, based on substrate binding and the architecture of the pocket, LJ0536 together with other bacterial feruloyl esterases, such as Est1E and CinI [35], should be clustered together in a new group of feruloyl/cinnamoyl esterases.

The functional role of cinnamoyl esterases in the host is a field of great interest as this kind of enzyme can be used as an additive to increase the nutritional value of foods of a vegetal origin. The structures and observations discussed in this study suggest further exploration of feruloyl/cinnamoyl esterases in other bacterial species could reveal further structural and functional diversity.

## Materials and Methods

### Chemicals

All chemicals were purchased from Sigma-Aldrich unless specified otherwise. Water was purified with Synergy® UV Millipore Water Purification System.

### Cloning, Expression, and Purification of LJ0536, LJ0536 Mutants, and Human Valacyclovirase (VACVase)

The LJ0536 p15TV-L clone [14] was used as a wild-type plasmid template for the mutations. The mutants were constructed by PCR using Phusion™ high fidelity DNA polymerase from Finnymes according to the manufacturer's protocol. The amino acids selected for mutagenesis were replaced with alanine. The 39-nucleotide long complementary primers containing the desired mutation in the middle of the primers were synthesized by Sigma-Aldrich®. The forward primer 5′-GGGCAACAATTGCCTATTTATGAA-3′ and reverse primer 5′GGGTCCTTGTGATTACCTTCAA′ were used for PCR amplification to generate a deletion mutant of the inserted α/β subdomain (coding region from Val_147_ to Ala_173_) The resulting PCR fragment was flanked by SmaI restriction sites. It was treated with T4 DNA ligase to seal the SmaI restriction site to complete the recombinant plasmid. The PCR-amplified plasmids were treated with 20 units of DpnI at 37°C for 2 hours to digest the methylated wild-type plasmid template. The plasmids containing the desired mutations were transformed into *E. coli* DH5α. Mutated sequences were confirmed by DNA sequencing. The plasmid containing the gene of interest (pET17b-VACVase) was provided by Dr. Gordon L. Amidon, University of Michigan. The gene of interest was cloned into p15TV-L vector according to the protocol described by Lorca et al. [36] and transformed into *Eschericha coli* DH5α. The sequence was confirmed by DNA sequencing. The expression of His_6_-tagged proteins was carried out using *E. coli* BL21-DE3 (Stratagene) with 1 mM IPTG (Isopropyl β-D-thiogalactopyranoside) as the inducer. Cells were disrupted by French Press and proteins were purified by nickel affinity chromatography as previously described [14] with the following modification. HEPES-based buffer (pH 7.50) instead of Tris-HCl based buffer was used throughout the purification process to improve the total yield of proteins. For removal of the His_6_-tag for enzymatic studies, purified proteins were incubated with TEV protease (60 µg TEV protease per 1 mg of target protein) at 4°C for 16 hours. The His_6_-tag was removed by passing the sample through a nickel affinity chromatography column. Collected proteins were dialyzed at 4°C against a buffer containing 50 mM HEPES pH 7.50, 500 mM NaCl, and 1 mM DTT (dithiothreitol) for 16 hours. Final protein products were flash-frozen and preserved in small aliquots at −80°C until use.

### Crystallization, Data Collection, and Structure Solution

All proteins (with His_6_-tag uncleaved) were crystallized using the sitting drop method with Intelliplate 96-well plates and a Mosquito Crystal liquid handling robot (TTP LabTech), mixing 0.5 µL of protein at 15 mg/mL, and 0.5 µL of reservoir solution, over 100 µL reservoir solution. The protein solutions were pre-treated with the proteases subtilisin and V8 for the apo and S106A mutant forms of the enzyme, respectively, and proteases stored at 1 mg/mL stock solution, added to final 1∶10 vol/vol ratio protease∶protein. Successful crystallization required the presence of the different proteases, a technique often used to increase the success of crystallization due to removal of disordered/flexible regions that would disrupt crystal formation [37].

Reservoir solutions were identified through an in-house custom crystallization screen that was optimized based on success of common commercial sparse-matrix crystallization screens [38] were as follows: apo enzyme: 0.1 M MES pH 6 and 20% PEG10K; S106A mutant: 0.1 M sodium cacodylate pH 6.5, 0.2 M calcium acetate, 9% PEG 8K; S106A co-crystallized with ethyl ferulate Form I: 0.1 M Tris pH 8.5, 0.2 M ammonium sulphate, 25% PEG 3350; S106A co-crystallized with ethyl ferulate Form II: 0.1 M Tris pH 8.5, 0.2 M ammonium sulphate, 24% PEG 3350; S106A co-crystallized with chlorogenic acid: 0.1 M Tris pH 8.5, 0.2 M lithium sulphate, 30% PEG 4K; S106A co-crystallized with ferulic acid: 0.1 M Tris pH 8.5, 0.2 M lithium sulfate, 30% PEG 4K.

Ligands were co-crystallized at a final ligand concentration of 5 mM (25 mM for S106A ethyl ferulate Form II) in the sitting drop, by diluting a stock solution of 100 mM ligand 1∶20 v/v with the protein/protease mix; 0.5 µL of this new solution was mixed with 0.5 µL of reservoir solution for crystallization.

All crystals were cryo-protected with reservoir solution supplemented with paratone-N oil [39] prior to flash freezing in an Oxford Cryosystems cryostream. Diffraction data at 100 K at the Cu-Kα wavelength were collected at the Structural Genomics Consortium using a Rigaku FR-E Superbright rotating anode with a Rigaku R-AXIS HTC detector. Diffraction data was reduced with HKL2000 [40].

The LJ0536 apo structure was solved by Molecular Replacement (MR) using Phaser [41], with a poly-alanine form of the structure of feruloyl esterase (Est1E, PDB:2WTM) from *Butyrivibrio proteoclasticus* [20] as a search model. The successful MR solution was identified by map inspection using Coot [42] and by a decrease in R_free_ after refinement using Refmac [43]. The structure was fully built by manual building and rounds of refinement with Refmac, Phenix.refine [44] and Buster [45] at the final stages. Anisotropic B-factors were refined for protein and ligand atoms for all structures. Non-crystallographic (NCS) restraints were not utilized for any structure. All structures were refined using TLS parameterization (TLS groups were the N-terminal residue to residue 179, and 180 to the C-terminal residue), as assigned by the TLSMD server [46]. Addition of TLS restraints resulted in lower R and R_free_ values. Water atoms were added by automatic methods using the refinement programs used in each structure (Phenix.refine, Refmac/CCP4/ARP/wARP, or BUSTER, respectively). Ions were added after the automatic water building by inspection of magnitude of residual F_o_-F_c_ density and hydrogen bonding patterns. The final atomic model includes residues 1–245 of LJ0536, with six atoms from the expression tag at the N-terminus of one chain of the asymmetric unit.

The LJ0536 S106A structure was solved by MR using the apo structure. All ligands were identified by the presence of residual F_o_-F_c_ density in the active site of the enzyme after molecular replacement using the apo S106A enzyme. Refinement of ligand structures was executed with geometric restraints generated by the PRODRG server [47] and with a combination of Refmac and/or Phenix.refine. Final validation of the structure of the ligands was performed by calculating simulated annealing omit F_o_-F_c_ maps using Phenix.refine and Cartesian simulated annealing with default parameters, after removing atoms from the ligand and any protein atoms within 5 Å of the ligand atoms.

In the LJ0536 S106A+ethyl ferulate Form I complex (two chains in the asymmetric unit), one ligand was modeled with an occupancy of 1.0 and the other with a manually-assigned occupancy of 0.55 (due to lower quality electron density, and higher B-factors than nearby protein atoms, at higher occupancy levels). For ethyl ferulate Form II complex (one chain in the asymmetric unit), the ligand was modeled with an occupancy of 1.0. All other ligands in their respective complexes were modeled with occupancies of 1.0.

All structures were refined until convergence of R_work_ and R_free_ values, and reasonable geometries were verified using the Procheck [48] and Molprobity [49] servers.

### PDB Coordinates

The structures of apo WT LJ0536, apo LJ0536 S106A, LJ0536 S106A+chlorogenic acid, LJ0536 S106A+ethyl ferulate Form I, LJ0536 S106A+ethyl ferulate Form II, and LJ0536 S106A+ferulic acid have been submitted to the PDB with the accession codes 3PF8, 3PF9, 3S2Z, 3PFB, 3QM1, and 3PFC, respectively.

### Structural Analysis

Protein-protein interaction interfaces were identified and analyzed with the PDBe PISA server [50] with default settings; a residue is considered in an interface if its change in accessible surface area between chain A and chain A complex with chain B is non-zero. All structural images were generated using PyMOL [51]. Structure similarity searches were performed using the Dali database [24].

### Enzymatic Assays

The esterase activities with aliphatic (4-nitrophenyl butyrate) and aromatic (ethyl ferulate, chlorogenic acid, and rosmarinic acid) substrates were measured spectrophotometrically using a Synergy HT Biotek Reader at 412 nm for aliphatic esters and 324 nm for aromatic esters. A typical reaction mixture contained 20 mM HEPES pH 7.80, 0.1 mM ester substrates, and 0.3 µg/mL purified enzymes (ethyl ferulate and chlorogenic acid) or 3 µg/mL purified enzymes (4-nitrophenyl butyrate and rosmarinic acid). The reactions were carried out at 25°C for mutated and wild-type LJ0536 and 37°C for VACVase. The extinction coefficients of 4-nitrophenyl butyrate (16300 M^−1^ cm^−1^), ethyl ferulate (15390 M^−1^ cm^−1^), chlorogenic acid (26322 M^−1^ cm^−1^), and rosmarinic acid (15670 M^−1^ cm^−1^) were used to determine the amount of substrate hydrolyzed. The assay was performed in triplicate and the percentage activity was calculated based on the average specific activity. Saturation kinetic assays were performed using the classical model substrate 4-nitrophenyl butyrate under the conditions previously described [14]. The kinetic parameters were estimated by non-linear fitting using Origin software (OriginLab, Northampton, MA).

### High Performance Liquid Chromatography (HPLC) Analysis

HPLC assays were performed using the Hitachi HPLC L-2000 series system with a Symmetry® C18 5 µm 3.9 mm×150 mm reversed-phase column and a Symmetry® C18 5 µm guard column. The determination of esterase activity using valacyclovir as the substrate was carried out as described by Lai *et al.* [28]. The reaction mixture contained 50 mM HEPES pH 7.80, 4 mM valacyclovir, and 10 µg/mL enzyme. The determination of esterase activities using ethyl ferulate, chlorogenic acid, and rosmarinic acid was carried out using linear gradient elution with water/acetic acid/1-butanol (350∶1∶7, vol∶vol∶vol) and methanol with a flow rate of 1 mL/min as described by Mastihuba et al. [52]. The reaction mixture contained 20 mM HEPES pH 7.80, 1 mM ester substrate, and 20 µg/mL enzyme.

### Fast Protein Liquid Chromatography (FPLC) Analysis

The relative molecular weights of proteins were determined using Pharmacia Biotech LCC-501 Plus FPLC System with a Superose 12 10/300GL column. Immunoglobulin G (150 kDa), bovine serum albumin (66 kDa), ovalbumin (45 kDa), trypsinogen (24 kDa), α-lactalbumin (14.2 kDa), cytochrome C (12.3 kDa), and vitamin B12 (1.4 kDa) were used as molecular weight standards, and 10 mM HEPES with 150 mM NaCl was used as the mobile phase.

### Circular Dichroism Assay

Protein secondary structure was estimated using AVIV Quick Start 215 Circular Dichroism Spectrometer with Hellma #110 Series 0.1 cm quartz cuvette. Protein samples at a concentration of 1.1 mg/mL were dialyzed against 2 mM HEPES buffer with 50 mM NaCl for 16 hours. Protein samples were adjusted to 0.2 mg/mL in 0.5 mM HEPES buffer with 10 mM NaCl after dialysis. Spectra were acquired at 1 nm intervals and averaged with 10 scans. Scans with buffer alone were used as background correction. The final spectra was expressed in molar ellipticity (ME) using the formula ME = θ/10nCl, where θ is the signal acquired, n is the number of residues, C is the molar concentration of protein, and l is the pathlength of the cuvette.

### Multiple Sequence Alignments of LJ0536 Structural Homologs

A structural similarity search was performed using the Dali server to identify proteins with structural homology. The sequences of structural homologs were retrieved from NCBI database [22]. Multiple sequence alignment was performed using CLUSTAL X2 [53].

## Supporting Information

Figure S1 **Multiple sequence alignment of LJ0536 with structural homologs.** Mutations were made on the residues in the rectangle. The catalytic triad is underlined. Stars indicate fully conserved residues. Colons represent semi-conserved residues. Only chain A is showed in the alignment. PDB 3PF8: LJ0536 (cinnamoyl esterase), *Lactobacillus johnsonii N6.2*. PDB 2WTM: EST1E (feruloyl esterase), *Butyrivibrio proteoclasticus*. PDB 3HJU: MAGL (monoglyceride lipase), human. PDB 3JW8: MGLL (monoglyceride lipase), human. PDB 1A8Q: CPO-A1 (chloroperoxidase A1), *Streptomyces aureofaciens*. PDB 1ZOI: EST (esterase), *Pseudomonas putida*. PDB 2OCG: VACVase (valacyclovir hydrolase), human.
(DOC)

Figure S2 **Relative position of the active (Ser_106_, His_225_, Asp_197_) and inactive (Ser_68_, His_32_, Asp_61_) catalytic triads of LJ0536.** The orientation of Ser_68_, His_32_, and Asp_61_ makes it impossible to form an active catalytic triad.
(TIF)

Figure S3 **Circular dichroism spectra of wild type LJ0536 and Ser68Ala in 0.5 mM HEPES buffer with 10 mM NaCl.** The overall structure of LJ0536 changed when Ser_68_ was mutated, supporting the importance of Ser_68_ on hydrogen bond formation to the central core of the protein in order to maintain the proper folding.
(TIF)

Figure S4 **Orientation of catalytic residues and structural superimposition of LJ0536 S106A co-crystallized with ferulic acid (FA) and ethyl ferulate (EF).** (A). Cartoon representation of LJ0536 S106A co-crystallized with ferulic acid and ethyl ferulate. LJ0536 S106A with ferulic acid bound is colored in light violet. LJ0536 S106A with ethyl ferulate bound is colored in yellow. Water molecules are represented by red spheres. The 4-hydroxyl group on the phenolic ring of ferulic acid and ethyl ferulate is hydrogen bonded with Asp_138_ in order to orient the phenolic ring in the correct position. Additional polar interactions of 4-hydroxyl and 3-methoxy groups with water molecules further stabilize the binding of substrate. The oxyanion hole is formed by Phe_34_ and Gln_107_. Gln_145_ positions a water molecule adjacent to the ester bond of substrate, which might be involved with the activation of Ser_106_. (B). Cutaway view of the LJ0536 S106A surface representation showing the phenolic ring binding pocket and the leaving groove.
(TIF)
