# Supplementary material for: An Inserted α/β Subdomain Shapes the Catalytic Pocket of Lactobacillus johnsonii Cinnamoyl Esterase
Source: PLoS One. 2011 Aug 18;6(8):e23269. doi: 10.1371/journal.pone.0023269 (PMC3158066; doi:10.1371/journal.pone.0023269)
Supplement: Figure S1 — Multiple sequence alignment of LJ0536 with structural homologs. Mutations were made on the residues in the rectangle. The catalytic triad is underlined. Stars indicate fully conserved residues. Colons represent semi-conserved residues. Only chain A is showed in the alignment. PDB 3PF8: LJ0536 (cinnamoyl esterase), Lactobacillus johnsonii N6.2. PDB 2WTM: EST1E (feruloyl esterase), Butyrivibrio proteoclasticus. PDB 3HJU: MAGL (monoglyceride lipase), human. PDB 3JW8: MGLL (monoglyceride lipase), human. PDB 1A8Q: CPO-A1 (chloroperoxidase A1), Streptomyces aureofaciens. PDB 1ZOI: EST (esterase), Pseudomonas putida. PDB 2OCG: VACVase (valacyclovir hydrolase), human. (DOC) [file pone.0023269.s001.doc]

Supplemental Fig.S1

**....|....| ....|....| ....|....| ....|....| ....|....| ....|....| ....|....|**

**10 20 30 40 50 60 70**

**LJ0536**  **NLYFQGMATI TLERDGLQLV GTREEPFGEI YDMAIIFHGF TANRNTSLLR EIANSLRDEN IASVRFDFNG**

**Est1E**  **-------GAM YIDCDGIKLN AYLDMPNPEK CPLCIIIHGF TGHSEERHIV AVQETLNEIG VATLRADMYG**

**MAGL**  **--------LP HLVNAGQYLF CRYWKPTGTP KALIFVSHGA GEHSGR--YE ELARMLMGLD LLVFAHDHVG**

**MGLL**  **--------LP HLVNAGQYLF CRYWKPTGTP KALIFVSHGA GEHSGR--YE ELARMLMGLD LLVFAHDHVG**

**CPO-A1**  **---------P ICTTDGVEIF YKDWG-QGRP --VV-FIHGW PLNGDA--WQ DQLKAVVDAG YRGIAHDRRG**

**EST**  **---------S YVTTDGVQIF YKDWGPRDAP --VIHFHHGW PLSADD--WD AQLLFFLAHG YRVVAHDRRG**

**VACVase**  **-----SVTSA KVAVNGVQLH YQQTG----E DHAVLLLPGM LGSGETD-FG PQLKNLNKKL FTVVAWDPRG**

**Clustal Consensus**  *** : . * . . * ***

**....|....| ....|....| ....|....| ....|....| ....|....| ....|....| ....|....|**

**80 90 100 110 120 130 140**

**LJ0536**  **HGDSDGKFEN MTVLNEIEDA NAILNYVKTD PHVRNIYLVG HSQGGVVASM LAGLYPDLIK KVVLLAPAAT**

**Est1E**  **HGKSDGKFED HTLFKWLTNI LAVVDYAKKL DFVTDIYMAG HSQGGLSVML AAAMERDIIK ALIPLSPAAM**

**MAGL**  **HGQSEGERMV VDFHVFVRDV LQHVDSMQKD YPGLPVFLLG HSMGGAIAIL TAAERPGHFA GMVLISPLVL**

**MGLL**  **HGQSEGERMV VDFHVFVRDV LQHVDSMQKD YPGLPVFLLG HSMGGAIAIL TAAERPGHFA GMVLISPLVL**

**CPO-A1**  **HGHSTPVWDG YDFDTFADDL NDLLTDLDL- ---RDVTLVA HSMGGGELAR YVGRGTGRLR SAVLLSAIPV**

**EST**  **HGRSSQVWDG HDMDHYADDV AAVVAHLG-- ----GAVHVG HSTGGGEVVR YMARHPEKVA KAVLIAAVPL**

**VACVase**  **YGHSRPDRDF -FFERDAKDA VDLMKALKFK ----KVSLLG WSDGGITALI AAAKYPSYIH KMVIWG----**

**Clustal Consensus** **:* * . : : . * ** . . : .**

**....|....| ....|....| ....|....| ....|....| ....|....| ....|....| ....|....|**

**150 160 170 180 190 200 210**

**LJ0536**  **LKGDALEGNT QGVTYNPDHI PDRLPFKDLT LGGFYLRIAQ QLPIYEVSAQ FTKPVCLIHG TDDTVVSPNA**

**Est1E**  **IPEIARTGEL LGLKFDPENI PDELDAWGRK LKGNYVRVAQ TIRVEDFVDK YTKPVLIVHG DQDEAVPYEA**

**MAGL**  **-ANPESATKV LAAKPNLSLG PAGLKVC--- ----FGIQLL NSRVERALPK LTVPFLLLQG SADRLCDSKG**

**MGLL**  **-ANPESATKV LAAKPNLSLG PAGLKVC--- ----FGIQLL NSRVERALPK LTVPFLLLQG SADRLCDSKG**

**CPO-A1**  **MIKPD---EV F-ALKNTERS -TIEGVR--- ----CVDAFG YTDFTEDLKK FDIPTLVVHG DDDQVVPIDT**

**EST**  **MVQPK---SV FDGFAQVA-A KAHYDG---- -----IVAFS QTDFTEDLKG IQQPVLVMHG DDDQIVPYES**

**VACVase**  **-ANAYVTDSM IYEGRDVSAR TCWVDG---- -----IRQFK GNICRHLLPR VQCPALIVHG EKDPLVPRFH**

**Clustal Consensus**  **. : * :::* ***

**....|....| ....|....| ....|....| ....|....| .**

**220 230 240 250**

**LJ0536**  **SKKYDQIYQN STLHLIEGAD HCFSDSYQKN AVNLTTDFLQ N**

**Est1E**  **SVAFSKQYKN CKLVTIPGDT HCYDHHLELV TEAVKEFMLE -**

**MAGL**  **AYLLMELAKD KTLKIYEGAY HVKELPVTNS VFHEINMWVS Q**

**MGLL**  **AYLLMELAKD KTLKIYEGAY HVKELPVTNS VFHEINMWVS Q**

**CPO-A1**  **GRKSAQIIPN AELKVYEGSS HGIVPGDKEK FNRDLLEFLN K**

**EST**  **GVLSAKLLPN GALKTYKGYP HGMPTTHADV INADLLAFIR S**

**VACVase**  **ADFIHKHVKG SRLHLMPEGK HNLHLRFADE FNKLAEDFLQ -**

**Clustal Consensus** **. : . * * :**
