# Supplementary figures and images for: An Inserted α/β Subdomain Shapes the Catalytic Pocket of Lactobacillus johnsonii Cinnamoyl Esterase
Source: PLoS One. 2011 Aug 18;6(8):e23269. doi: 10.1371/journal.pone.0023269 (PMC3158066; doi:10.1371/journal.pone.0023269)

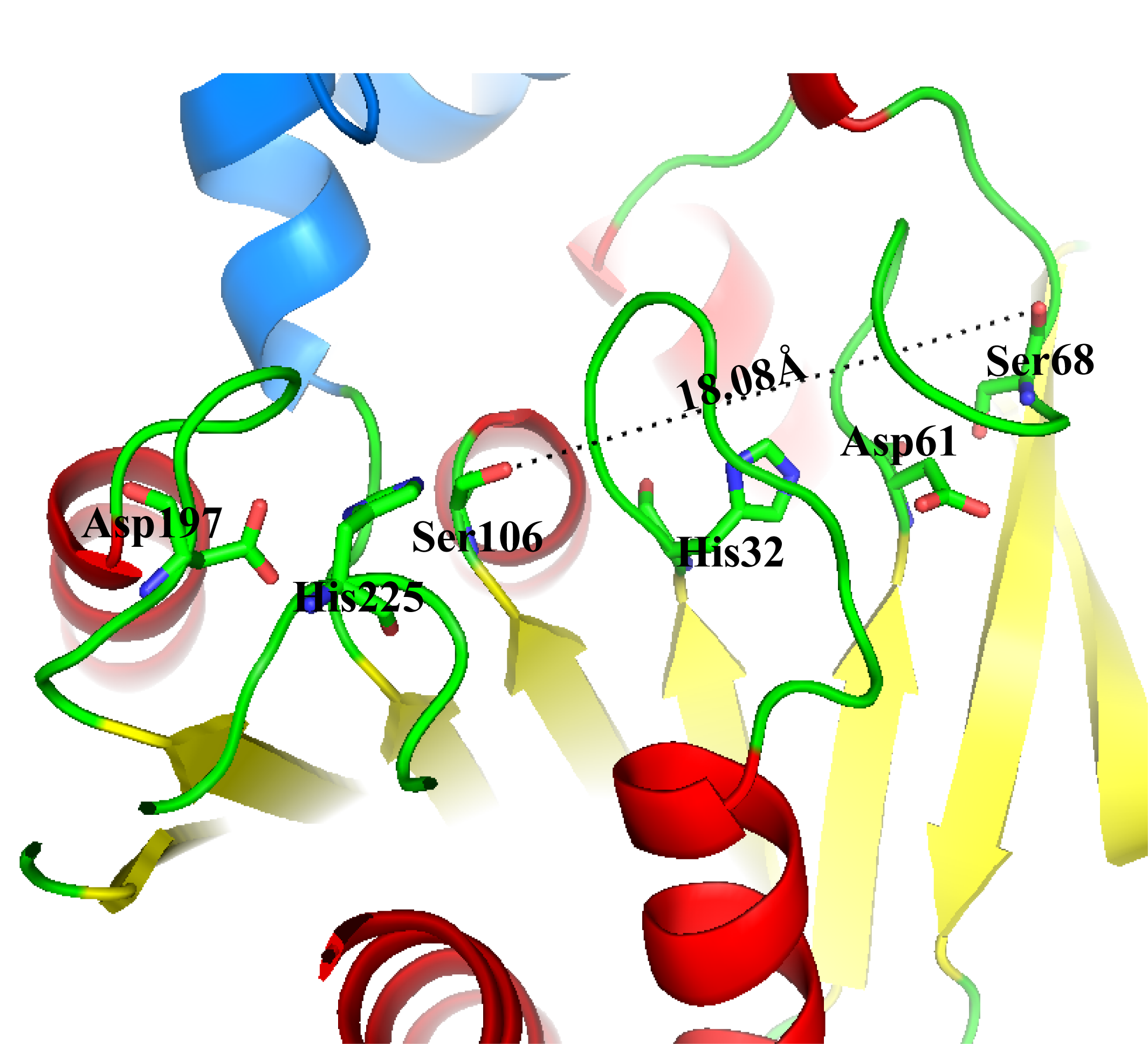

Supplement: Figure S2 — Relative position of the active (Ser106, His225, Asp197) and inactive (Ser68, His32, Asp61) catalytic triads of LJ0536. The orientation of Ser68, His32, and Asp61 makes it impossible to form an active catalytic triad. (TIF) [file pone.0023269.s002.tif]

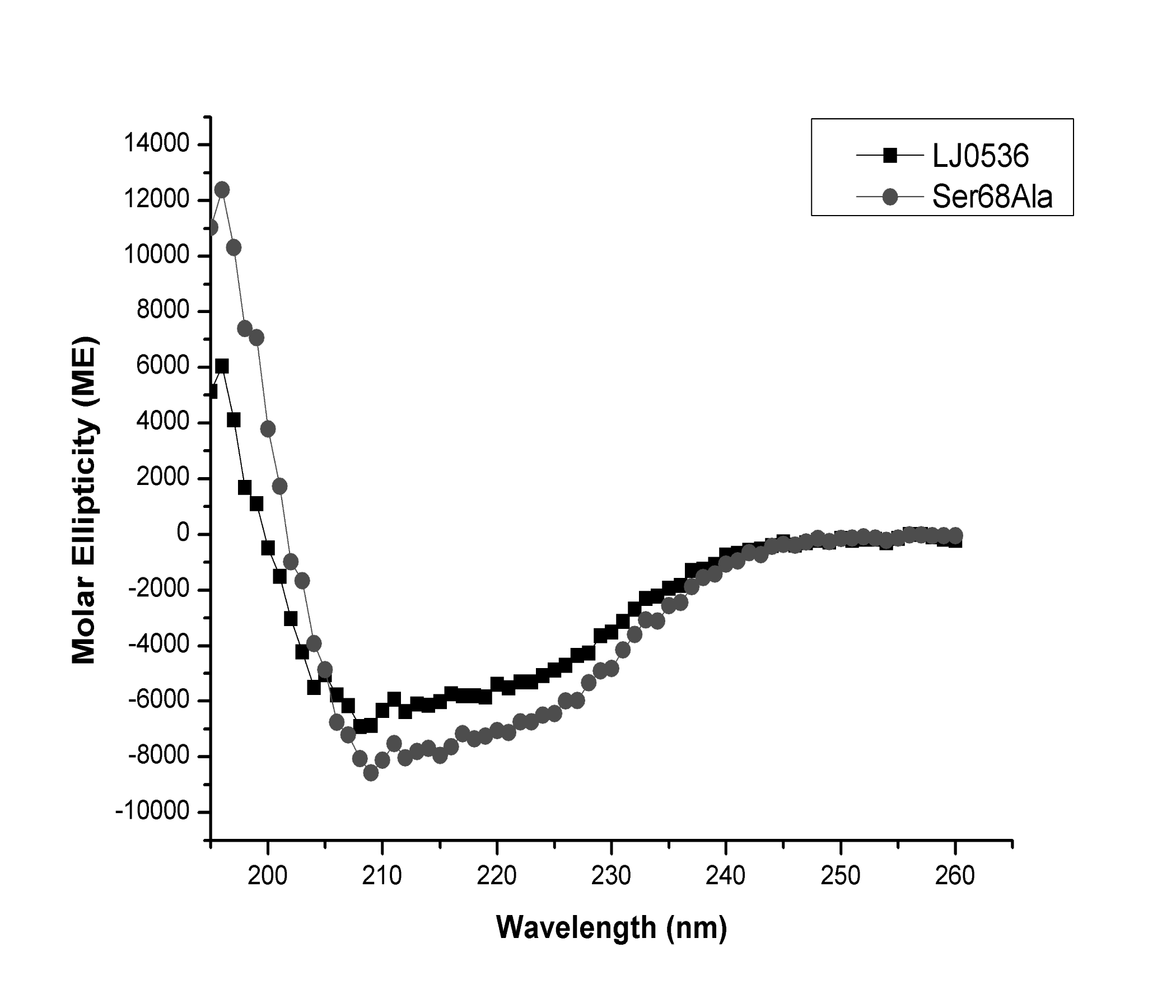

Supplement: Figure S3 — Circular dichroism spectra of wild type LJ0536 and Ser68Ala in 0.5 mM HEPES buffer with 10 mM NaCl. The overall structure of LJ0536 changed when Ser68 was mutated, supporting the importance of Ser68 on hydrogen bond formation to the central core of the protein in order to maintain the proper folding. (TIF) [file pone.0023269.s003.tif]

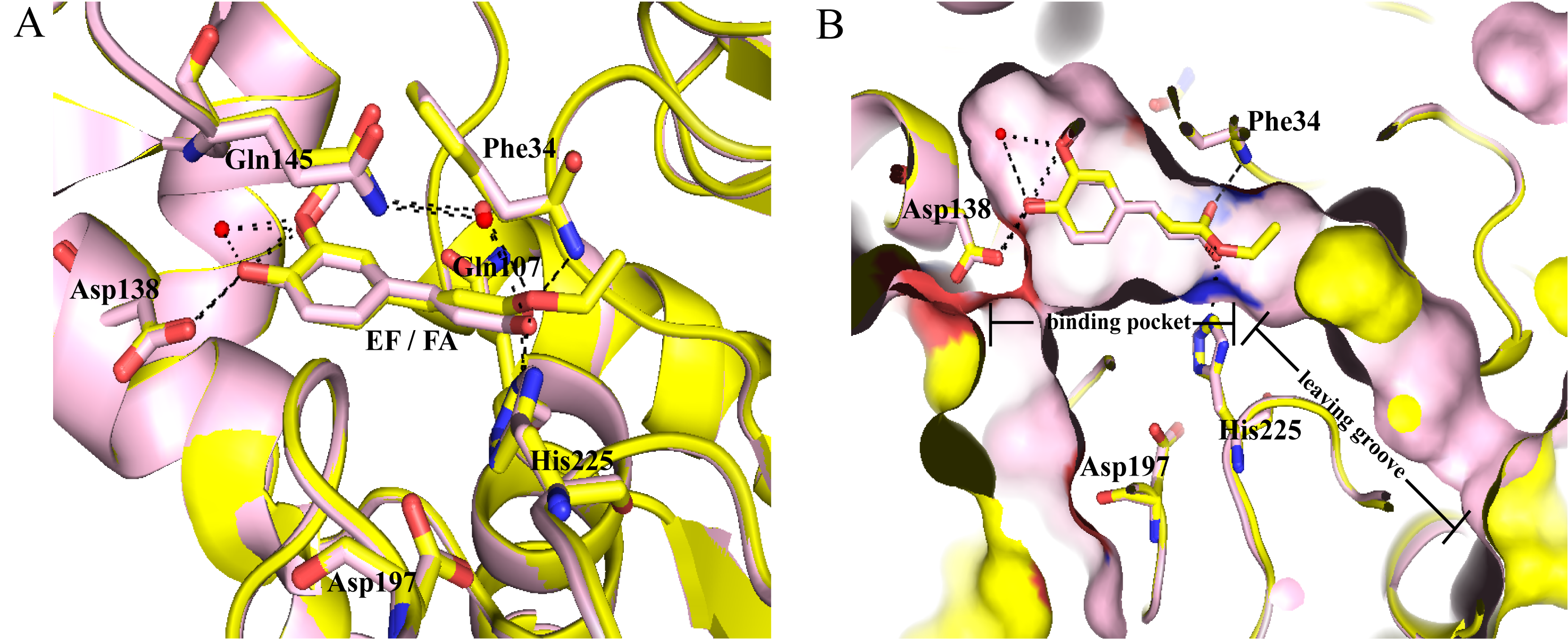

Supplement: Figure S4 — Orientation of catalytic residues and structural superimposition of LJ0536 S106A co-crystallized with ferulic acid (FA) and ethyl ferulate (EF). (A). Cartoon representation of LJ0536 S106A co-crystallized with ferulic acid and ethyl ferulate. LJ0536 S106A with ferulic acid bound is colored in light violet. LJ0536 S106A with ethyl ferulate bound is colored in yellow. Water molecules are represented by red spheres. The 4-hydroxyl group on the phenolic ring of ferulic acid and ethyl ferulate is hydrogen bonded with Asp138 in order to orient the phenolic ring in the correct position. Additional polar interactions of 4-hydroxyl and 3-methoxy groups with water molecules further stabilize the binding of substrate. The oxyanion hole is formed by Phe34 and Gln107. Gln145 positions a water molecule adjacent to the ester bond of substrate, which might be involved with the activation of Ser106. (B). Cutaway view of the LJ0536 S106A surface representation showing the phenolic ring binding pocket and the leaving groove. (TIF) [file pone.0023269.s004.tif]
